# Exploring the differential effects of psychological resilience and social support in mitigating post-traumatic psychiatric symptoms: real-world network analysis of front-line rescuers

**DOI:** 10.1192/bjo.2024.64

**Published:** 2024-05-10

**Authors:** Peng Cheng, Lirong Wang, Ying Zhou, Wenjing Ma, Weihui Li

**Affiliations:** Department of Psychiatry, National Clinical Research Center for Mental Disorders, and National Center for Mental Disorders, The Second Xiangya Hospital of Central South University, China; Department of Thyroid Surgery, The Xiangya Hospital of Central South University, China

**Keywords:** Psychological resilience, social support, network analysis, cross-sectional study, post-traumatic stress disorder (PTSD)

## Abstract

**Background:**

Although both psychological resilience and social support are widely believed to be effective in alleviating post-traumatic psychiatric symptoms in individuals with traumatic events, there has been a lack of comparative analysis of their intervention effects on different post-traumatic psychiatric symptoms. Furthermore, previous studies have mostly failed to control for potential confounding effects caused by different traumatic events.

**Aims:**

We used the novel network analysis approach to examine the differential moderating effects of psychological resilience and social support on post-traumatic psychiatric symptoms, controlling for the confounding effects of traumatic events.

**Method:**

We recruited 264 front-line rescuers who experienced the same traumatic event. Quantified edge weights and bridge expected influence (BEI) were applied to compare the alleviating effects of psychological resilience and social support.

**Results:**

Our study revealed distinct correlations in a sample of front-line rescuers: social support negatively correlates more with psychosomatic symptoms, notably fatigue in depressive networks and sleep disturbance in post-traumatic stress disorder (PTSD) networks, whereas psychological resilience shows fewer such correlations. Quantitative analysis using BEI indicated that psychological resilience more effectively suppresses depressive and anxiety symptom networks, whereas social support more significantly inhibits PTSD symptom networks.

**Conclusions:**

The current study represents the first attempt to examine the differential effects of psychological resilience and social support on post-traumatic outcomes in real-world emergency rescuers, controlling for the confounding effect of traumatic events. Our results can act as the theoretical reference for future precise and efficient post-trauma psychological interventions.

Individuals who have experienced exposure to a traumatic event have a significantly higher risk of subsequent comorbid mental disorders, including post-traumatic stress disorder (PTSD), anxiety and depression,^1^ which significantly reduces their overall quality of life.^2^ Thus, investigating factors that may be protective for mental health outcomes is important. Previous studies have identified two major categories of protective factors:^3^ ‘subjective’ (e.g. individual background and characteristics such as resilience) and ‘objective’ (e.g. external intervention and social support).

## Psychological resilience and social support

Psychological resilience refers to an individual's ability to adapt and recover from adversity, stress, trauma or tragedy. It involves not avoiding challenges, but maintaining normal functioning amid difficulties, and the potential for personal growth and development through such experiences.^4^ Research conducted across various samples has consistently shown a negative correlation between psychological resilience and psychiatric symptoms following exposure to traumatic events.^5,6^ Moreover, these symptoms encompass not only PTSD symptoms, but also symptoms of depression and anxiety. This indicates that psychological resilience acts as a protective factor against various negative mental health outcomes following exposure to traumatic events.^7^

Social support encompasses a spectrum of assistance and resources provided by an individual's social network, including emotional, informational, material and companionship support. This multifaceted support, originating from sources such as family, friends, colleagues and professional groups, is instrumental in reducing stress and improving quality of life. It includes not just tangible help and advice, but also the emotional and social companionship essential for well-being.^8^ Similar to psychological resilience, previous research has also indicated a negative correlation between social support and post-traumatic psychiatric symptoms in individuals exposed to traumatic events. Specifically, higher levels of social support can reduce the severity of PTSD symptoms.^9,10^ Furthermore, social support can reduce the risk of depression and anxiety symptoms, potentially by enhancing individuals’ perception of life's meaning and reducing feelings of loneliness.^11,12^

## Shortcomings of existing studies

This literature suggests that psychological resilience and social support can reduce the severity or risk of psychiatric symptoms after traumatic events. Although a direct causal relationship cannot be asserted, psychological resilience and social support have been widely demonstrated to be significant factors that can alleviate post-traumatic psychiatric symptoms. However, to our knowledge, few studies have focused on the differences in the efficacy of psychological resilience and social support in mitigating post-traumatic psychiatric symptoms. Most research to date has identified correlations between either psychological resilience or social support and post-traumatic psychiatric symptoms within their respective cohorts,^9,13^ lacking a quantifiable approach to explore the differential effects of psychological resilience and social support on these symptoms.

From a methodological standpoint, existing research on the effects of psychological resilience and social support on psychiatric symptoms post-trauma has several limitations. First, the impact of psychological resilience and social support may differ among individuals who have experienced various types of traumatic events, rendering comparisons across diverse traumatic event samples less reliable. Second, in light of the symptom network theory, which posits that interactions between symptoms can contribute to the development and persistence of mental disorders,^14^ most traditional analyses employing the latent variable approach focus on the influence of psychological resilience or social support on overall mental disorders. This approach lacks a symptom-oriented analysis, potentially overlooking intricate relationships at the symptom level,^15^ thus failing to provide a comprehensive understanding of these effects.

## The objectives and hypotheses of this study

This study utilises network analysis, a novel symptom-based analysis approach to address this research gap. The principle of network analysis is to represent the structure of mental disorder as a network, in which nodes are symptoms and edges represent the strength of the relationship or influence between them. Additionally, as a symptom-oriented approach, network analysis provides a way to quantify and measure the importance of individual nodes and edges within the network.^16^ In the context of network analysis, the centrality of each node can be quantified to assess the significance of the node within the network.^17^ In this study, we utilised the bridging centrality index to evaluate the influence of psychological resilience and social support on PTSD, anxiety and depression symptoms. This approach was selected to enable a direct comparison of the efficacy of these two factors in ameliorating post-traumatic psychological symptoms.^18^ Additionally, to minimise potential confounding factors (such as trauma type, occupation background, etc.) and accurately assess the differential effects of psychological resilience and social support on post-traumatic psychiatric symptoms, only individuals with exposure to the same traumatic event were recruited in our sample.

To the best of our knowledge, the current study represents the first attempt to recruit a sample of individuals who have experienced the same traumatic event in the real world, and utilise a robust network analysis method to compare the effect of psychological resilience and social support on post-traumatic psychological symptoms. Concerned about the inherent heterogeneity of psychological resilience and social support in their attributes, we hypothesise that they may also exhibit differential effects on the alleviation of distinct post-traumatic psychiatric symptom clusters. Demonstrating the effects of psychological resilience and social support on different clusters of post-traumatic psychiatric symptoms within the real world is beneficial for providing more efficient and personalised psychological interventions, which can lead to better treatment outcomes.

## Method

### Study design and participants

The current study is a cross-sectional study conducted in Changsha, Hunan province, China. The participants in our study consisted exclusively of front-line rescuers involved in the ‘4.29’ building collapse accident, a significant building collapse accident that occurred on 29 April 2023 in Changsha, Hunan, China, resulting in 54 fatalities and ten injuries. This incident garnered substantial attention from the Chinese Government and its highest-ranking officials. These front-line rescuers, all members of fire brigades under the jurisdiction of the Changsha Municipal People's Government, had received comprehensive disaster response training, including building collapse scenarios, as part of their routine preparedness drills. This specialised training provided them with emergency rescue skills. In response to the incident, they were quickly dispatched by the municipal government to the site to initiate rescue efforts. Our survey was conducted approximately 1 month after the incident, specifically between June and July 2023. The reason for choosing this period for the survey is because the DSM-5 criteria for PTSD diagnosis require that the relevant symptoms persist for more than a month, and it also serves to differentiate these symptoms from those of acute stress disorder.

To improve efficiency in information collection and ensure data reliability, this study employed a web-based survey approach. Each participant, upon entering the link to our electronic survey questionnaire, was first required to review an informed consent document. This document outlined the objectives and details of our survey, which focused on assessing the psychological states of rescuers post-exposure to traumatic events, and delineated the usage of the collected data (solely for scientific research purposes, ensuring strict confidentiality of any personal privacy-related information). The participants proceeded with the survey of post-traumatic psychiatric symptoms only after they had electronically signed the consent form, thereby indicating their agreement and comprehension of the research purpose and details. In the absence of such consent, participants were automatically withdrawn from the survey process.

The inclusion criteria were as follows: (a) age above 18 years, (b) possession of a national firefighter certification, (c) engagement in front-line rescue operations during the ‘4.29’ building collapse accident and (d) voluntary participation in the survey. Exclusion criteria were as follows: (a) failure to work on the front line of the rescue during the accident (e.g. participating in logistical support work) or (b) failure to participate in the entire rescue operation during the accident for any reason (e.g. exiting after only participating in part of the rescue work).

To ensure a uniform level of exposure among the rescue personnel to the traumatic event, thereby achieving control over the traumatic event, this study conducted a screening of the rescuer sample based on their exposure at the accident rescue site. This screening process involved the nature of tasks undertaken during the rescue operation and the duration of time spent at the rescue site. Only those rescue workers who were engaged in personnel rescue tasks (in direct contact with the victims) rather than other logistical roles, and who participated in the rescue efforts for the entire duration from the start to the end of the rescue activities, were included in the subsequent analysis of this study.

Our study was approved by the Ethics Committee of the Second Xiangya Hospital of Central South University (approval number: LYG2022009). All respondents provided electronic written informed consent.

### Measures

Our study involved five distinct measures. The ten-item Connor–Davidson Resilience Scale (CD-RISC-10) and the Social Support Rating Scale (SSRS) were employed to assess psychological resilience and social support, respectively. For post-traumatic psychiatric symptoms, we used the Posttraumatic Stress Disorder Checklist for DSM-5 (PCL-5), the Patient Health Questionnaire-9 (PHQ-9) and the Generalised Anxiety Disorder-7 (GAD-7), to evaluate PTSD, depression and anxiety symptoms, respectively.

All of the scales used in this study were Chinese versions, which have been previously validated for their psychometric properties in research. Detailed information about each scale is provided in the following sections.

#### The CD-RISC-10

The CD-RISC-10 is a ten-item self-report measurement, which is widely applied to assess psychological resilience, and specifically the ability to deal with adversity.^19^ Higher total scores reflect a greater ability to cope with adversity. The reliability and validity of the Chinese version of the CD-RISC-10 have been established across diverse Chinese populations.^20^ In this study, the Cronbach's alpha coefficient for the CD-RISC-10 was 0.992.

#### The SSRS

The SSRS, compiled by Shui Yuan Xiao, is currently widely used to measure social support, and has good reliability and validity among the Chinese population.^21^ Comprising three dimensions – subjective social support (four items), objective social support (three items) and utilisation of support (three items) – the SSRS functions as a composite measure of total social support, with higher scores reflecting greater levels of social support. The Cronbach's alpha coefficient for the SSRS was 0.992 in the current study.

#### The PCL-5

The PCL-5 is a widely used self-report measure employed to evaluate PTSD symptoms among individuals who have experienced traumatic events. It comprises 20 items that correspond to the DSM-5 diagnostic criteria for PTSD, including intrusive thoughts, avoidance, negative alterations in cognition and mood, and hyperarousal symptoms.^22^ The PCL-5 is a reliable and valid assessment of PTSD symptoms in a variety of populations,^23^ including the Chinese population.^24^ A higher score on the PCL-5 indicates more severe symptoms of PTSD. According to previous research, the cut-off value for the PCL-5 has been established at 33 points in our research. A total score of 33 or above on the PCL-5 indicates a probable diagnosis of PTSD. The Cronbach's alpha coefficient for the PCL-5 was 0.955.

#### The PHQ-9

The PHQ-9 is a self-report questionnaire based on DSM-IV criteria, and is widely used for screening and assessing the severity of depressive symptoms. It is a four-point Likert scale questionnaire, with a score range of 0–3 for each item. The severity of depression can be assessed by calculating the total score, with higher scores reflecting greater levels of depression. The Chinese version of the PHQ-9 has been validated and demonstrated to be a reliable instrument for assessing depression within the general Chinese population.^25^ Based on prior research, the cut-off value for the PHQ-9 in this study has been set at 7 points. A score of 7 or higher on the PHQ-9 suggests a possible diagnosis of depression.^25^ The Cronbach's alpha coefficient for the PCL-5 was 0.927.

#### The GAD-7

The GAD-7 is a validated screening tool that consists of seven items designed to assess symptoms of anxiety. The individuals are required to indicate the frequency with which they have experienced each symptom described in the item statement, using a four-point Likert scale with options ranging from zero (not at all) to three (nearly every day). The psychometric property of the GAD-7 has been extensively demonstrated in Chinese populations.^26^ According to previous research findings, the cut-off value for the GAD-7 in this study has been established at 7 points. A score of 7 or higher on the GAD-7 indicates a potential diagnosis of anxiety disorder. The Cronbach's alpha coefficient for the PCL-5 was 0.905.

### Data analysis

Our research used the R programme for data analysis (R version 4.3.2 for Windows, R Core Team, Vienna, Austria; see https://www.R-project.org). The network analysis procedure was divided into three distinct domains: network estimation and visualisation, bridge centrality analysis, and estimation of network accuracy and stability.

#### Network estimation and visualisation

To compare the moderating effects of psychological resilience and social support on post-traumatic symptoms (i.e. PTSD, anxiety and depression), we constructed three distinct networks and conducted statistical analyses on each network. Specifically, both psychological resilience and social support were separately linked to three different symptom clusters – PTSD, anxiety and depression – to establish the three distinct networks.

In each network, the edges between any two nodes represent the partial correlation coefficients between them, while controlling for confounding effects from other nodes. Partial correlation coefficients are derived using multivariate linear regression models. This entails establishing regression models to predict each node, computing the residuals after adjusting for other nodes, and then assessing the correlation between these residuals. The outcome of this process represents the partial correlation coefficient between two target nodes, accounting for the influence of other nodes.

The extended Bayesian information criterion (EBIC) and the least absolute shrinkage and selection operator (LASSO) were used to refine the construction of network edges. The LASSO method applies regularisation penalties to partial correlation coefficients, effectively reducing the weights of statistically insignificant edges, thus promoting network sparsity. This process aids in eliminating noise and highlighting statistically significant edges, resulting in a more concise and interpretable network. EBIC was employed for model selection, imposing penalties on models with an excess of edges to balance model complexity with data fit. EBIC achieves this balance by adjusting the hyperparameter *γ*, with higher *γ*-values tending to produce sparser networks and reduce the likelihood of overfitting. In this study, following previous network analysis tutorials, the hyperparameter *γ* was set to 0.5.^27^ This combined approach of LASSO and EBIC allows researchers to enhance network accuracy as well as filtering out edges likely caused by random noise, producing a network model that is both robust and interpretable.^16^

Following the Fruchterman–Reingold algorithm, nodes with high centrality were strategically placed in more central positions within the network graph, demonstrating their stronger connections to other nodes. Various plotting features were employed to effectively illustrate the network's characteristics, with green edges representing positive associations and red edges depicting negative associations. Thicker and more vivid edges indicated stronger and closer relationships.^28^ The steps mentioned were implemented with the *bootnet* (version 1.5) and *qgraph* (version 1.9.2) R packages.

#### Bridge centrality analysis

Bridge expected influence (BEI) measures the significance of a node in connecting two distinct communities within a network. Nodes with a high BEI serve as pivotal connectors between two different node communities. A higher BEI indicates a greater probability of activating the opposite community, whereas a lower BEI suggests a heightened likelihood of inhibiting the opposite community.^28^ Therefore, this study uses the calculation of BEI to discern the varying effects of psychological resilience and social support on the alleviation of different types of post-traumatic psychiatric symptoms (PTSD, depressive and anxiety symptoms) within networks. The BEI is calculated as the sum of the edge weights between a given node and all nodes in the opposite community. Compared with the bridge strength, which is obtained by summing the absolute values of edge weights, using BEI as a centrality index in network analysis is more appropriate, particularly when the correlation between nodes is not clear.^29^

To facilitate the calculation of BEI, the communities to which nodes belong were predefined based on their attributes in the network. Because psychological resilience and social support both act as mitigating factors for post-traumatic psychiatric symptoms, they were grouped into the same community, which we have termed the ‘psychological protection’ community. Subsequently, in the three distinct networks of our study, the BEI was calculated between the ‘psychological protection’ community and the ‘PTSD symptoms’ community, the ‘depression symptoms’ community and the ‘anxiety symptoms’ community. The BEI was calculated by the function bridge of the R package *networktools* (version 1.4.0).

#### Estimation of network accuracy and stability

The estimation of network accuracy and stability encompassed the following two parts: the accuracy of edge weights and the differences of edge weights. To evaluate the accuracy of edge weights, a non-parametric bootstrapping method was employed to construct a 95% confidence interval, where a narrower interval indicates a more reliable estimation. As a means of evaluating the statistical differences in pairwise edge weights, bootstrapped difference tests based on 95% confidence intervals were conducted. When the value of zero is not present within the confidence intervals of either pairwise edge weights, it can be inferred that there exists a statistically significant difference between the two compared elements.^27^

## Results

### Descriptive information

As per our inclusion and exclusion criteria, a cohort of 264 competent front-line rescuers were recruited for the study. Details of the demographic characteristics and symptom assessments of the sample are shown in Table 1. From a demographic perspective, the sample population in this study consisted solely of men (100%), with the largest age group being 26–30 years (36.4%). It should be acknowledged that there is 9.8% missing data in the age variable of the sample. However, since the original data matrix used for the subsequent network analysis did not involve the age variable, the missing information on the age variable did not affect the results of the subsequent network analysis.

**Table 1:** Demographic information and psychiatric symptom assessment status

Category	*n* (%)
Gender	
Male	264 (100%)
Age (years)^a^	
19–25	90 (34.1%)
26–30	96 (36.4%)
31–40	44 (16.7%)
≥41	8 (3.0%)
Psychological resilience	27.90 ± 12.90
Social support	45.64 ± 9.07
PCL-5	
Intrusion (mean ± s.d.)	1.56 ± 2.12
Avoidance (mean ± s.d.)	0.37 ± 0.84
Negative mood/cognitions (mean ± s.d.)	1.33 ± 2.78
Hyperarousal (mean ± s.d.)	1.04 ± 1.92
Total score (mean ± s.d.)	4.30 ± 6.95
Probable PTSD diagnosis	4 (1.5%)
PHQ-9	
Total score (mean ± s.d.)	0.98 ± 2.74
Probable depression diagnosis	13 (4.9%)
GAD-7	
Total score (mean ± s.d.)	0.78 ± 2.18
Probable anxiety diagnosis	9 (3.4%)

PCL-5, Posttraumatic Stress Disorder Checklist for DSM-5; PTSD, post-traumatic stress disorder; PHQ-9, Patient Health Questionnaire-9; GAD-7, Generalised Anxiety Disorder-7.

a. 9.8% missing data in the age variable.

Results from the symptom screening indicate that probable depression was the most prevalent in the sample (4.9%), followed by probable anxiety (3.4%) and probable PTSD (1.5%). Overall, the incidence of potential PTSD, depression or anxiety among the front-line rescuers in our study was relatively low. Considering previous research also indicates that not all rescuers exposed to traumatic events develop psychiatric symptoms, with the majority not exhibiting any psychological symptoms,^30,31^ our findings are largely consistent with these prior studies. Additionally, detailed information on abbreviations, mean scores and standard deviations for each node of the corresponding network is shown in Table 2.

**Table 2:** Descriptive information of variables in the network analysis

Category	Abbreviation	Mean	s.d.
Psychological resilience		28.34	11.61
Social support		45.64	9.07
PTSD symptoms (PCL-5)			
Intrusive thoughts	P1	0.28	0.51
Nightmares	P2	0.18	0.46
Flashbacks	P3	0.17	0.43
Emotional cue reactivity	P4	0.68	0.75
Physical cue reactivity	P5	0.24	0.48
Avoidance of thoughts	P6	0.20	0.49
Avoidance of reminders	P7	0.17	0.42
Trauma-related amnesia	P8	0.28	0.58
Negative beliefs	P9	0.14	0.39
Distorted blame	P10	0.15	0.40
Persistent negative emotional state	P11	0.19	0.41
Lack of interest	P12	0.14	0.40
Feeling detached	P13	0.16	0.41
Inability to experience positive emotions	P14	0.13	0.39
Irritable/ angry	P15	0.15	0.41
Recklessness	P16	0.09	0.33
Hypervigilance	P17	0.20	0.44
Exaggerated state	P18	0.16	0.41
Difficulty concentrating	P19	0.25	0.53
Sleep disturbance	P20	0.33	0.64
Depressive symptoms (PHQ-9)			
Anhedonia	D1	0.12	0.40
Depressed or sad mood	D2	0.09	0.32
Sleep difficulties	D3	0.21	0.53
Fatigue	D4	0.19	0.48
Appetite changes	D5	0.11	0.38
Feeling of worthlessness	D6	0.08	0.35
Concentration difficulties	D7	0.08	0.32
Psychomotor agitation/retardation	D8	0.07	0.35
Thoughts of death	D9	0.03	0.24
Anxiety symptoms (GAD-7)			
Nervousness or anxiety	A1	0.12	0.41
Uncontrollable worry	A2	0.11	0.38
Worry too much	A3	0.15	0.45
Trouble relaxing	A4	0.15	0.44
Restlessness	A5	0.09	0.37
Irritable	A6	0.12	0.37
Afraid something will happen	A7	0.05	0.28

PTSD, post-traumatic stress disorder; PCL-5, Posttraumatic Stress Disorder Checklist for DSM-5; PHQ-9, Patient Health Questionnaire-9; GAD-7, Generalised Anxiety Disorder-7.

### Psychological resilience-social support-PTSD symptom network

**Fig. 1:**
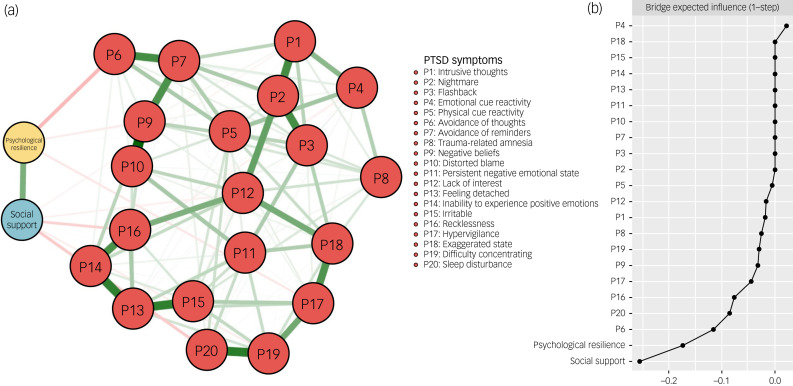
The network structure of the psychological resilience-social support-PTSD symptom network. (a) Symptom nodes with stronger associations are placed closer to each other. The dark green lines represent positive correlations. The red lines represent negative correlations. The line thickness represents the strength of the connection between symptom nodes. (b) Centrality plot represents the bridge expected influence of each node in the network. PTSD, post-traumatic stress disorder.

The network structure of the psychological resilience-social support-PTSD symptom network is shown in Fig. 1(a). Initially, 231 edges (22 × (22−1)/2) and 22 nodes were estimated, and then 118 non-zero edges were included for further analysis after selection of the LASSO algorithm. The psychological resilience-P6 (avoidance of thoughts) (weight = −0.12) and psychological resilience-P17 (hypervigilance) (weight = −0.04) edges showed the strongest associations between psychological resilience and PTSD symptom nodes, whereas the social support-P20 (sleep disturbance) (weight = −0.09) and social support-P16 (recklessness) (weight = −0.08) edges showed the strongest associations between social support and PTSD symptom nodes. Details are shown in Supplementary Table 1 available at https://doi.org/10.1192/bjo.2024.64. The BEI of social support (BEI of social support = −0.26) was found to be stronger than that of psychological resilience (BEI of psychological resilience = −0.17) (Fig. 1(b)). The accuracy of edge weights was confirmed by a bootstrapped test, as demonstrated in Supplementary Fig. 1. Additionally, the difference test of edge weights is shown in Supplementary Fig. 2.

### The psychological resilience-social support-depressive symptom network

**Fig. 2:**
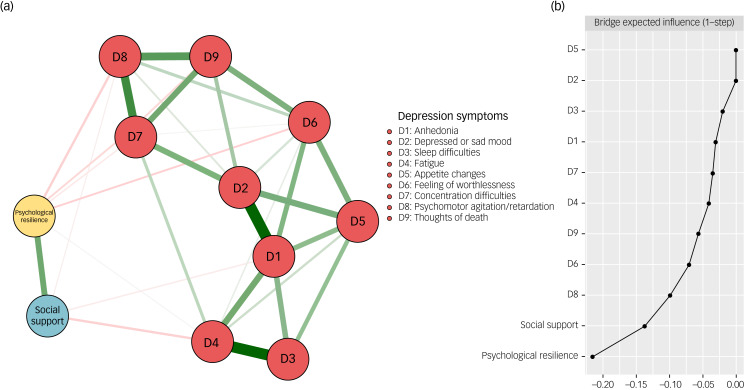
The network structure of the psychological resilience-social support-depressive symptom network. (a) Symptom nodes with stronger associations are placed closer to each other. The dark green lines represent positive correlations. The red lines represent negative correlations. The line thickness represents the strength of the connection between symptom nodes. (b) Centrality plot represents the bridge expected influence of each node in the network.

Fig. 2(a) illustrates the structure of the psychological resilience-social support-depressive symptom network. Initially, the LASSO algorithm estimated 55 edges (11 × (11−1)/2) and 11 nodes, out of which 34 non-zero edges were eligible for subsequent analysis. Among the connections between psychological resilience and depressive symptoms, the strongest negative edges with psychological resilience were observed for D6 (feeling of worthlessness) (weight = −0.07) and D8 (psychomotor agitation/retardation) (weight = −0.07). Nevertheless, the negative edges between D4 (fatigue) (weight = −0.05) and D1 (anhedonia) (weight = −0.03) with social support were the strongest among the connections between depressive symptoms and social support. Further details are presented in Supplementary Table 2. Fig. 2(b) shows the BEIs of the psychological resilience-social support-depressive symptom network. Both psychological resilience and social support had a relieving effect on depressive symptoms, with psychological resilience exhibiting a stronger relieving effect (BEI of psychological resilience = −0.22) compared with social support (BEI of social support = −0.14). The bootstrapped 95% confidence interval of edge weights, depicted in Supplementary Fig. 3, suggests that the accuracy of network edge weights was acceptable. The bootstrapped difference test of edge weights is presented in Supplementary Fig. 4.

### The psychological resilience-social support-anxiety symptom network

**Fig. 3:**
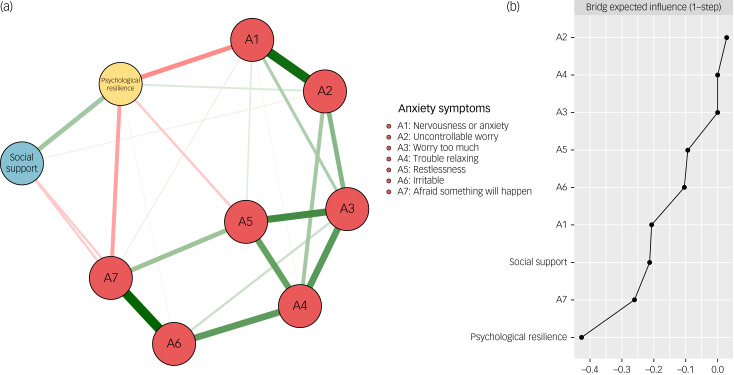
The network structure of the psychological resilience-social support-anxiety symptom network (a) Symptom nodes with stronger associations are placed closer to each other. The dark green lines represent positive correlations. The red lines represent negative correlations. The line thickness represents the strength of the connection between symptom nodes. (b) Centrality plot represents the bridge expected influence of each node in the network.

The structure of the psychological resilience-social support-anxiety symptom network is depicted in Fig. 3(a). Initially, 36 edges (9 × (9–1)/2) and nine nodes were estimated, followed by the implementation of the LASSO algorithm, which identified 25 non-zero edges for further analysis. The edge weight matrix (Supplementary Table 3) revealed that the most prominent negative edges between psychological resilience and anxiety symptom nodes were psychological resilience-A1 (nervousness or anxiety) (weight = −0.21) and psychological resilience-A7 (afraid something will happen) (weight = −0.21). Regarding the edges linking social support and anxiety symptoms, the strongest associations were observed between A7 (afraid something will happen) (weight = 0.10) and A6 (irritable) (weight = 0.08) with social support. Similar to the psychological resilience-social support-depressive symptom network, the BEI of psychological resilience (BEI of psychological resilience = −0.43) was stronger than that of social support (BEI of social support = −0.21) (Fig. 3(b)). The accuracy of edge weight estimates was verified through a bootstrapped accuracy test (Supplementary Fig. 5). The bootstrapped difference test of edge weights is demonstrated in Supplementary Fig. 6.

## Discussion

To our knowledge, our study is the first to employ the emerging and reliable network analysis method in the real world to investigate and compare the alleviating effects of psychological resilience and social support on symptoms of depression, anxiety and PTSD. Importantly, this study controlled for the type and severity of traumatic events experienced by the respondents, and therefore provides a more rigorous examination of the differences in the effects of psychological resilience and social support on the alleviation of post-traumatic psychiatric symptoms in a sample of male front-line rescuers, compared with previous studies that used samples with varying types and severity of trauma exposure. The findings of this study can be mainly divided into two parts. First, using network visualisation (represented as edge weights in the network structure), we analysed the associations between psychological resilience, social support and different symptom nodes in various subnetworks. Second, BEI was used as a measure to horizontally compare the differences in the relieving effects of psychological resilience and social support in depressive, anxiety and PTSD symptom subnetworks.

Although psychological resilience and social support have negative weight edges with many nodes in the post-traumatic psychiatric symptom networks, there were still differences in their correlations with specific symptom nodes, such as psychosomatic symptom nodes. Social support shows more negative correlations with psychosomatic symptom nodes; for instance, social support-fatigue (in the depressive symptom network) and social support-sleep disturbance (in the PTSD symptom network). However, psychological resilience rarely shows negative correlations with psychosomatic symptom nodes.

Considering that in the three networks, both social support and psychological resilience have very low BEIs, this suggests that both factors are inversely correlated with post-traumatic psychiatric symptoms and exhibit a high level of inhibitory effect on the post-traumatic psychiatric symptom networks. Additionally, given that social support has established many negative-weighted edges with psychosomatic symptom nodes, we speculate that the inhibitory effect of social support on the activation of psychosomatic symptoms following trauma may be more significant than that of psychological resilience.

Previous research has demonstrated the association between social support and both fatigue^32,33^ and sleep disturbance^34^ across various populations. There are several reasonable interpretations for the result. First, compared with psychological resilience, social support is not only a form of psychological assistance, but also includes objective material conditions (such as a better resting environment and adequate nutritional support). This is particularly important for front-line rescuers who have just experienced a severe disaster, as it can help them alleviate a series of common post-disaster psychosomatic symptoms, including fatigue and sleep disorders. Second, individuals situated within supportive environments are more inclined to adopt behavioural modifications aimed at mitigating psychosomatic symptoms.^35^ For instance, a supportive partner can indirectly enhance an emergency rescuer's sleep hygiene. This contribution is not through direct monitoring, but rather by creating an environment conducive to rest, which can diminish sleep disturbance. However, it must be acknowledged that, although previous studies have indicated a positive ameliorative effect of social support on fatigue and sleep disturbances, its impact on psychosomatic symptoms may still vary among individuals.

Because of the quantified reflection of the BEI on the overall impact of nodes on their connected opposite-side networks, our study indicates that, in a sample of front-line rescuers, psychological resilience plays a more significant role than social support in suppressing the activation of networks associated with depressive and anxiety symptoms. However, the converse is true for networks of PTSD symptoms, where social support exhibits a more pronounced inhibitory effect on activation than psychological resilience. These findings suggest that the impacts of psychological resilience and social support on mental health symptom networks differ.

We suggest that the results of this study provide theoretical reference for future accurate and efficient interventions for individuals in front-line rescuer roles. Specifically, interventions targeting individuals predominantly afflicted with depression and anxiety should prioritise enhancing psychological resilience, such as through cognitive–behavioural therapy.^36^ Conversely, for those primarily experiencing PTSD symptoms, the focus should be on bolstering social support, such as by establishing social support groups.^37^ Considering the limited mental healthcare resources in China, formulating practical and efficient intervention strategies is important for effectively mitigating post-traumatic psychiatric symptoms with minimal resource expenditure.

Although the current research demonstrated the difference between psychological resilience and social support in alleviating post-traumatic psychiatric symptoms, there are still some limitations worth mentioning. First, given that post-traumatic psychiatric symptoms are influenced by a multitude of factors, and although psychological resilience and social support are two important elements among them, gathering information on other variables that may affect post-traumatic psychiatric symptoms and using them as covariates to adjust research findings can enhance the reliability of the results. Therefore, it is essential for future research to validate the findings of this study based on the collection of additional information on variables potentially related to post-traumatic psychiatric symptoms. Second, although the study employed objective measures to control exposure to the traumatic event, it did not systematically and quantifiably assess the participants’ subjective experiences. This limits the generalisability of the findings. Future research should integrate objective exposure levels with standardised subjective assessments, to more effectively control for trauma exposure in sample populations. Third, despite the fact that the self-report questionnaires applied in our research have been widely adopted with reliable psychometric properties, the network structures might still be affected by recall bias or the concealment of psychiatric symptoms due to the stigma. Fourth, the cross-sectional study design restricted the investigation of network dynamic alterations and causality directions. Additionally, all of the networks built in our cross-sectional research reveal the between-individual effects from the perspective of the group level, rather than the within-person level. Future longitudinal network analysis for the specific network variation within an individual is necessary to ascertain the alleviation effect trajectory of psychological resilience and social support on post-traumatic psychiatric symptoms.

Fifth, the sample included in this study comprised exclusively male front-line rescuers, thereby limiting its representativeness and the generalisability of our findings. Given the potential differences in post-traumatic psychiatric symptoms between genders^38^ and the higher level of disaster preparedness among front-line rescuers compared with the general population, it is necessary to validate our study's conclusions in future research involving female front-line rescuers and members of the public exposed to disasters in non-professional capacities.

In summary, the current study represents the first attempt to examine the differential effects of psychological resilience and social support on post-traumatic outcomes in real-world emergency rescuers, while controlling for the confounding effect of traumatic events. Specifically, we found that social support is more effective than psychological resilience in alleviating somatic symptoms after the traumatic event, and has a greater effect on reducing PTSD symptoms compared with psychological resilience. In contrast, psychological resilience outperforms social support in ameliorating anxiety and depression symptoms following trauma. Our results can act as the theoretical reference for future precise and efficient post-trauma psychological interventions for individuals who have experienced traumatic events.

## Supporting information

Cheng et al. supplementary material Cheng et al. supplementary material

## Data Availability

The data that support the findings of this study are available from the corresponding author, W.L., on reasonable request.
