# Supplementary material for: Exploring the differential effects of psychological resilience and social support in mitigating post-traumatic psychiatric symptoms: real-world network analysis of front-line rescuers
Source: BJPsych Open. 2024 May 10;10(3):e109. doi: 10.1192/bjo.2024.64 (PMC11094433; doi:10.1192/bjo.2024.64)
Supplement: Cheng et al. supplementary material [file S2056472424000644sup001.docx]

**Supplementary materials**

**Supplementary Table S1** Weights of edges between nodes in the psychological resilience-social support-PTSD symptom network

**Supplementary Table S2** Weights of edges between nodes in the psychological resilience-social support-depressive symptom network

**Supplementary Table S3** Weights of edges between nodes in the psychological resilience-social support-anxiety symptom network

**Supplementary Figure S1** Bootstrapped confidence intervals of edge weights of the psychological resilience-social support-PTSD symptom network

**Supplementary Figure S2** Estimation of edge weight difference by bootstrapped difference test of the psychological resilience-social support-PTSD symptom network

**Supplementary Figure S3** Bootstrapped confidence intervals of edge weights of the psychological resilience-social support-depressive symptom network

**Supplementary Figure S4** Estimation of edge weight difference by bootstrapped difference test of the psychological resilience-social support- depressive symptom network

**Supplementary Figure S5** Bootstrapped confidence intervals of edge weights of the psychological resilience-social support-anxiety symptom network

**Supplementary Figure S6** Estimation of edge weight difference by bootstrapped difference test of the psychological resilience-social support- anxiety symptom network

|  | P1 | P2 | P3 | P4 | P5 | P6 | P7 | P8 | P9 | P10 | P11 | P12 | P13 | P14 | P15 | P16 | P17 | P18 | P19 | P20 | PR | SS |
| --- | --- | --- | --- | --- | --- | --- | --- | --- | --- | --- | --- | --- | --- | --- | --- | --- | --- | --- | --- | --- | --- | --- |
| P1 | 0.00 | 0.31 | 0.12 | 0.19 | 0.01 | 0.00 | 0.11 | 0.04 | 0.02 | 0.00 | 0.00 | 0.00 | 0.00 | 0.00 | 0.00 | 0.00 | 0.00 | 0.00 | 0.00 | 0.00 | 0.00 | -0.02 |
| P2 | 0.31 | 0.00 | 0.30 | 0.00 | 0.06 | 0.02 | 0.14 | 0.00 | 0.00 | 0.00 | 0.00 | 0.25 | -0.01 | 0.00 | -0.02 | 0.00 | 0.00 | 0.00 | 0.00 | 0.01 | 0.00 | 0.00 |
| P3 | 0.12 | 0.30 | 0.00 | 0.03 | 0.05 | 0.03 | 0.07 | 0.06 | 0.11 | 0.00 | 0.13 | 0.00 | 0.00 | 0.02 | 0.00 | 0.00 | 0.03 | 0.12 | 0.01 | 0.01 | 0.00 | 0.00 |
| P4 | 0.19 | 0.00 | 0.03 | 0.00 | 0.15 | 0.03 | 0.04 | 0.13 | 0.00 | 0.00 | 0.00 | 0.00 | -0.01 | -0.03 | 0.00 | -0.01 | 0.02 | 0.00 | 0.00 | 0.01 | 0.02 | 0.00 |
| P5 | 0.01 | 0.06 | 0.05 | 0.15 | 0.00 | 0.14 | 0.13 | 0.08 | 0.00 | 0.07 | 0.04 | 0.00 | 0.02 | 0.00 | 0.07 | 0.00 | 0.00 | 0.09 | 0.00 | 0.08 | -0.01 | 0.00 |
| P6 | 0.00 | 0.02 | 0.03 | 0.03 | 0.14 | 0.00 | 0.30 | 0.00 | 0.04 | 0.04 | 0.00 | 0.01 | 0.00 | 0.03 | 0.00 | 0.00 | 0.00 | 0.00 | 0.00 | 0.00 | -0.12 | 0.00 |
| P7 | 0.11 | 0.14 | 0.07 | 0.04 | 0.13 | 0.30 | 0.00 | 0.00 | 0.29 | 0.00 | 0.00 | 0.00 | 0.00 | 0.00 | 0.00 | 0.01 | 0.04 | 0.00 | 0.01 | 0.04 | 0.00 | 0.00 |
| P8 | 0.04 | 0.00 | 0.06 | 0.13 | 0.08 | 0.00 | 0.00 | 0.00 | 0.07 | 0.01 | 0.00 | 0.08 | 0.02 | 0.00 | 0.06 | 0.00 | 0.00 | 0.06 | 0.00 | 0.00 | 0.00 | -0.03 |
| P9 | 0.02 | 0.00 | 0.11 | 0.00 | 0.00 | 0.04 | 0.29 | 0.07 | 0.00 | 0.41 | 0.00 | 0.03 | 0.00 | 0.11 | 0.00 | 0.09 | 0.00 | 0.00 | 0.00 | 0.00 | -0.03 | 0.00 |
| P10 | 0.00 | 0.00 | 0.00 | 0.00 | 0.07 | 0.04 | 0.00 | 0.01 | 0.41 | 0.00 | 0.14 | 0.11 | 0.07 | 0.00 | 0.00 | 0.04 | 0.00 | 0.03 | 0.00 | 0.00 | 0.00 | 0.00 |
| P11 | 0.00 | 0.00 | 0.13 | 0.00 | 0.04 | 0.00 | 0.00 | 0.00 | 0.00 | 0.14 | 0.00 | 0.00 | 0.11 | 0.03 | 0.08 | 0.00 | 0.01 | 0.13 | 0.09 | 0.09 | 0.00 | 0.00 |
| P12 | 0.00 | 0.25 | 0.00 | 0.00 | 0.00 | 0.01 | 0.00 | 0.08 | 0.03 | 0.11 | 0.00 | 0.00 | 0.01 | 0.10 | 0.05 | 0.21 | 0.02 | 0.22 | 0.00 | 0.00 | 0.00 | -0.02 |
| P13 | 0.00 | -0.01 | 0.00 | -0.01 | 0.02 | 0.00 | 0.00 | 0.02 | 0.00 | 0.07 | 0.11 | 0.01 | 0.00 | 0.34 | 0.34 | 0.14 | 0.10 | 0.00 | 0.07 | 0.00 | 0.00 | 0.00 |
| P14 | 0.00 | 0.00 | 0.02 | -0.03 | 0.00 | 0.03 | 0.00 | 0.00 | 0.11 | 0.00 | 0.03 | 0.10 | 0.34 | 0.00 | 0.06 | 0.29 | 0.01 | 0.00 | 0.00 | 0.00 | 0.00 | 0.00 |
| P15 | 0.00 | -0.02 | 0.00 | 0.00 | 0.07 | 0.00 | 0.00 | 0.06 | 0.00 | 0.00 | 0.08 | 0.05 | 0.34 | 0.06 | 0.00 | 0.06 | 0.13 | 0.00 | 0.10 | 0.01 | 0.00 | 0.00 |
| P16 | 0.00 | 0.00 | 0.00 | -0.01 | 0.00 | 0.00 | 0.01 | 0.00 | 0.09 | 0.04 | 0.00 | 0.21 | 0.14 | 0.29 | 0.06 | 0.00 | 0.00 | 0.00 | 0.00 | 0.00 | 0.00 | -0.08 |
| P17 | 0.00 | 0.00 | 0.03 | 0.02 | 0.00 | 0.00 | 0.04 | 0.00 | 0.00 | 0.00 | 0.01 | 0.02 | 0.10 | 0.01 | 0.13 | 0.00 | 0.00 | 0.29 | 0.23 | 0.01 | -0.04 | 0.00 |
| P18 | 0.00 | 0.00 | 0.12 | 0.00 | 0.09 | 0.00 | 0.00 | 0.06 | 0.00 | 0.03 | 0.13 | 0.22 | 0.00 | 0.00 | 0.00 | 0.00 | 0.29 | 0.00 | 0.08 | 0.01 | 0.00 | 0.00 |
| P19 | 0.00 | 0.00 | 0.01 | 0.00 | 0.00 | 0.00 | 0.01 | 0.00 | 0.00 | 0.00 | 0.09 | 0.00 | 0.07 | 0.00 | 0.10 | 0.00 | 0.23 | 0.08 | 0.00 | 0.35 | 0.00 | -0.03 |
| P20 | 0.00 | 0.01 | 0.01 | 0.01 | 0.08 | 0.00 | 0.04 | 0.00 | 0.00 | 0.00 | 0.09 | 0.00 | 0.00 | 0.00 | 0.01 | 0.00 | 0.01 | 0.01 | 0.35 | 0.00 | 0.00 | -0.09 |
| PR | 0.00 | 0.00 | 0.00 | 0.02 | -0.01 | -0.12 | 0.00 | 0.00 | -0.03 | 0.00 | 0.00 | 0.00 | 0.00 | 0.00 | 0.00 | 0.00 | -0.04 | 0.00 | 0.00 | 0.00 | 0.00 | 0.21 |
| SS | -0.02 | 0.00 | 0.00 | 0.00 | 0.00 | 0.00 | 0.00 | -0.03 | 0.00 | 0.00 | 0.00 | -0.02 | 0.00 | 0.00 | 0.00 | -0.08 | 0.00 | 0.00 | -0.03 | -0.09 | 0.21 | 0.00 |

**Supplementary Table S1** Weights of edges between nodes in the psychological resilience-social support-PTSD symptom network

|  | D1 | D2 | D3 | D4 | D5 | D6 | D7 | D8 | D9 | PR | SS |
| --- | --- | --- | --- | --- | --- | --- | --- | --- | --- | --- | --- |
| D1 | 0.00 | 0.40 | 0.19 | 0.23 | 0.18 | 0.20 | 0.00 | 0.00 | 0.00 | 0.00 | -0.03 |
| D2 | 0.40 | 0.00 | 0.01 | 0.00 | 0.21 | 0.07 | 0.20 | 0.06 | 0.14 | 0.00 | 0.00 |
| D3 | 0.19 | 0.01 | 0.00 | 0.40 | 0.16 | 0.00 | 0.00 | 0.00 | 0.00 | 0.00 | -0.02 |
| D4 | 0.23 | 0.00 | 0.40 | 0.00 | 0.08 | 0.04 | 0.11 | 0.00 | 0.00 | 0.02 | -0.06 |
| D5 | 0.18 | 0.21 | 0.16 | 0.08 | 0.00 | 0.25 | 0.00 | 0.00 | 0.00 | 0.00 | 0.00 |
| D6 | 0.20 | 0.07 | 0.00 | 0.04 | 0.25 | 0.00 | 0.02 | 0.10 | 0.20 | -0.07 | 0.00 |
| D7 | 0.00 | 0.20 | 0.00 | 0.11 | 0.00 | 0.02 | 0.00 | 0.30 | 0.22 | -0.04 | 0.00 |
| D8 | 0.00 | 0.06 | 0.00 | 0.00 | 0.00 | 0.10 | 0.30 | 0.00 | 0.26 | -0.07 | -0.03 |
| D9 | 0.00 | 0.14 | 0.00 | 0.00 | 0.00 | 0.20 | 0.22 | 0.26 | 0.00 | -0.06 | 0.00 |
| PR | 0.00 | 0.00 | 0.00 | 0.02 | 0.00 | -0.07 | -0.04 | -0.07 | -0.06 | 0.00 | 0.23 |
| SS | -0.03 | 0.00 | -0.02 | -0.06 | 0.00 | 0.00 | 0.00 | -0.03 | 0.00 | 0.23 | 0.00 |

**Supplementary Table S2** Weights of edges between nodes in the psychological resilience-social support-depressive symptom network

|  | A1 | A2 | A3 | A4 | A5 | A6 | A7 | PR | SS |
| --- | --- | --- | --- | --- | --- | --- | --- | --- | --- |
| A1 | 0.00 | 0.43 | 0.14 | 0.02 | 0.06 | 0.00 | -0.04 | -0.21 | 0.00 |
| A2 | 0.43 | 0.00 | 0.22 | 0.16 | 0.00 | 0.01 | 0.00 | 0.06 | -0.04 |
| A3 | 0.14 | 0.22 | 0.00 | 0.30 | 0.34 | 0.09 | 0.00 | 0.00 | 0.00 |
| A4 | 0.02 | 0.16 | 0.30 | 0.00 | 0.28 | 0.29 | 0.00 | 0.00 | 0.00 |
| A5 | 0.06 | 0.00 | 0.34 | 0.28 | 0.00 | 0.01 | 0.18 | -0.09 | 0.00 |
| A6 | 0.00 | 0.01 | 0.09 | 0.29 | 0.01 | 0.00 | 0.46 | -0.02 | -0.08 |
| A7 | -0.04 | 0.00 | 0.00 | 0.00 | 0.18 | 0.46 | 0.00 | -0.16 | -0.10 |
| PR | -0.21 | 0.06 | 0.00 | 0.00 | -0.09 | -0.02 | -0.16 | 0.00 | 0.17 |
| SS | 0.00 | -0.04 | 0.00 | 0.00 | 0.00 | -0.08 | -0.10 | 0.17 | 0.00 |

**Supplementary Table S3** Weights of edges between nodes in the psychological resilience-social support-anxiety symptom network

**
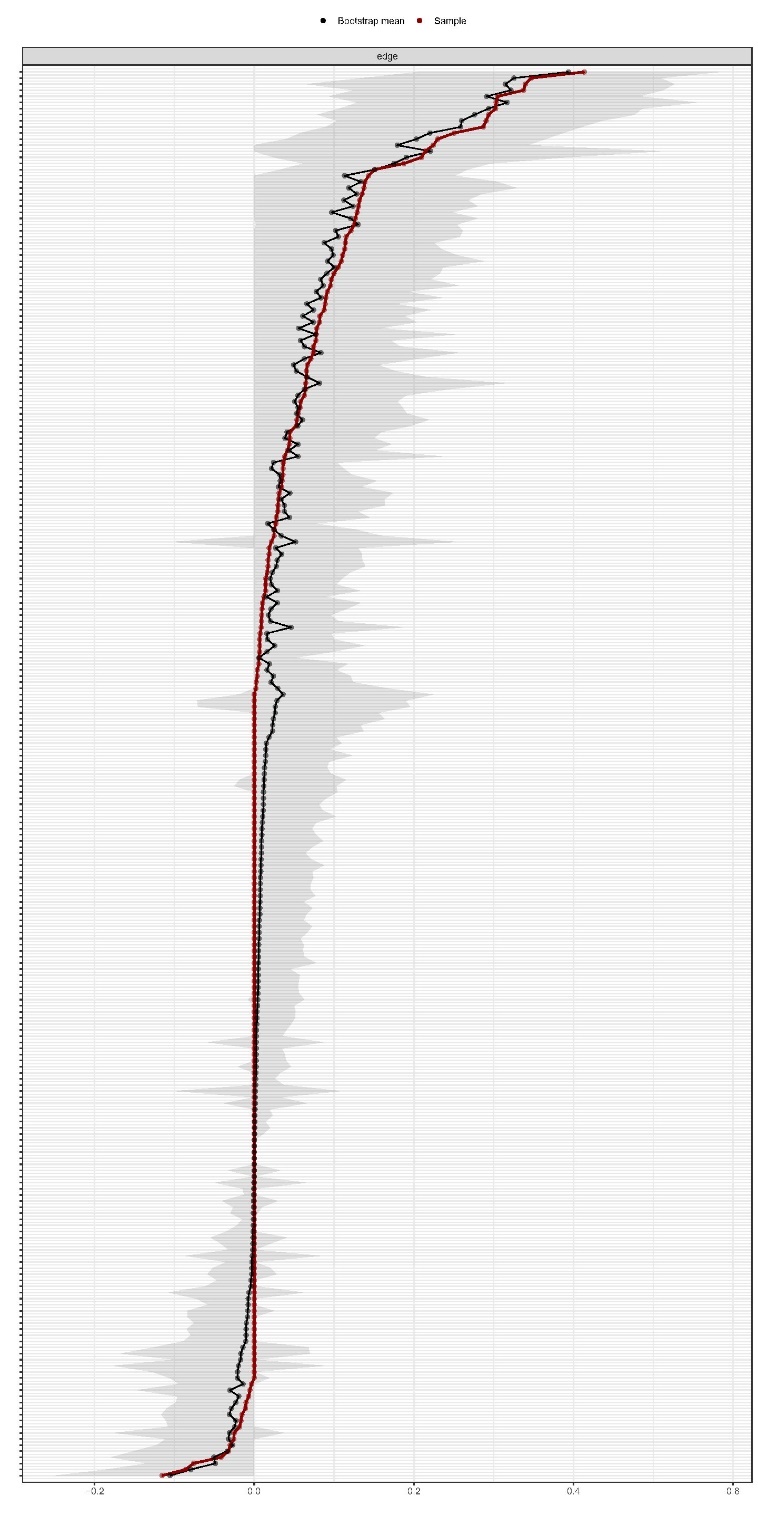
**

**Supplementary Figure S1** Bootstrapped confidence intervals of edge weights of the psychological resilience-social support-PTSD symptom network

The black dots indicate the values of each edge weight, ordered from the highest to the lowest value. The gray area represents the 95% Confidence Intervals of edge weights, estimated with the non-parametric bootstrap procedure (by R *bootnet* package). Wide intervals suggest lower stability and narrow intervals mean higher stability.


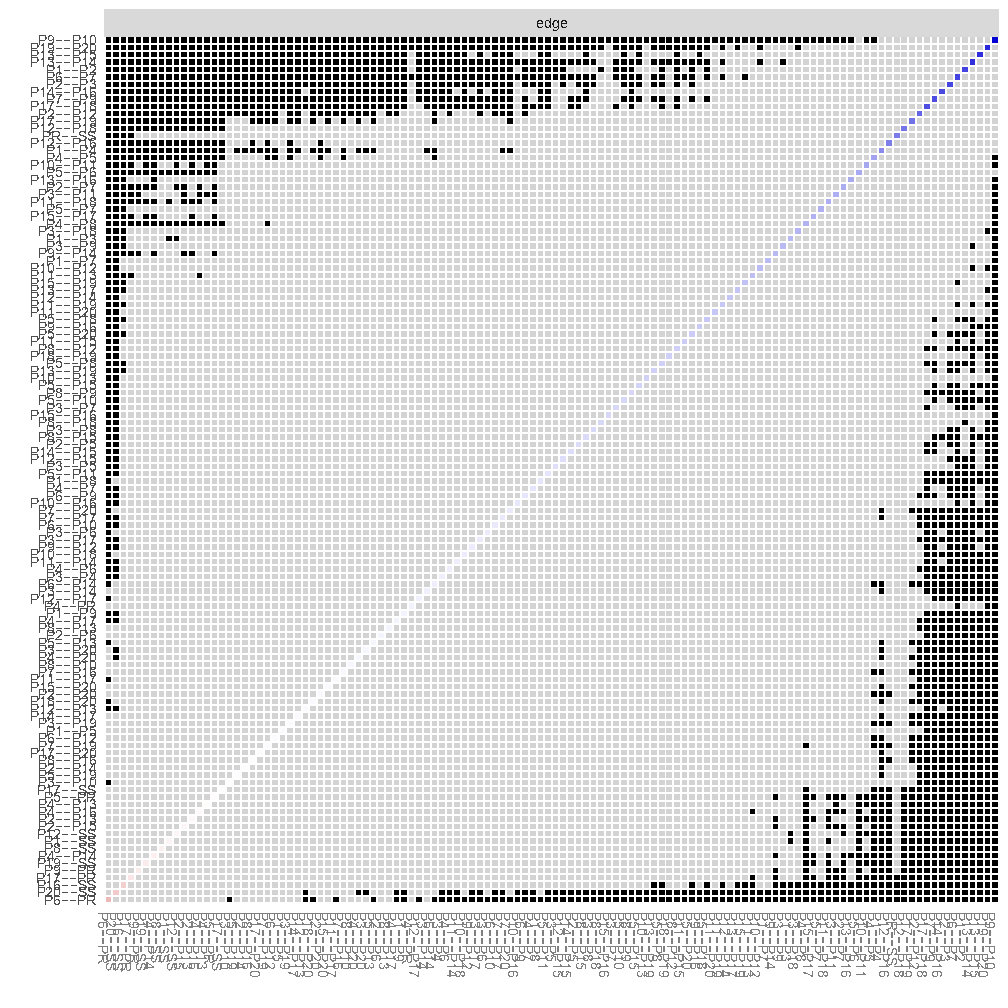


**Supplementary Figure S2.** Estimation of edge weight difference by bootstrapped difference test

Bootstrapped difference tests between edge weights in the network. Gray boxes suggest edges that do not differ significantly from one-another. Black boxes indicate edges with significant difference from one another (α = 0.05). Blue boxes in the edge-weight diagram indicate positive correlations.


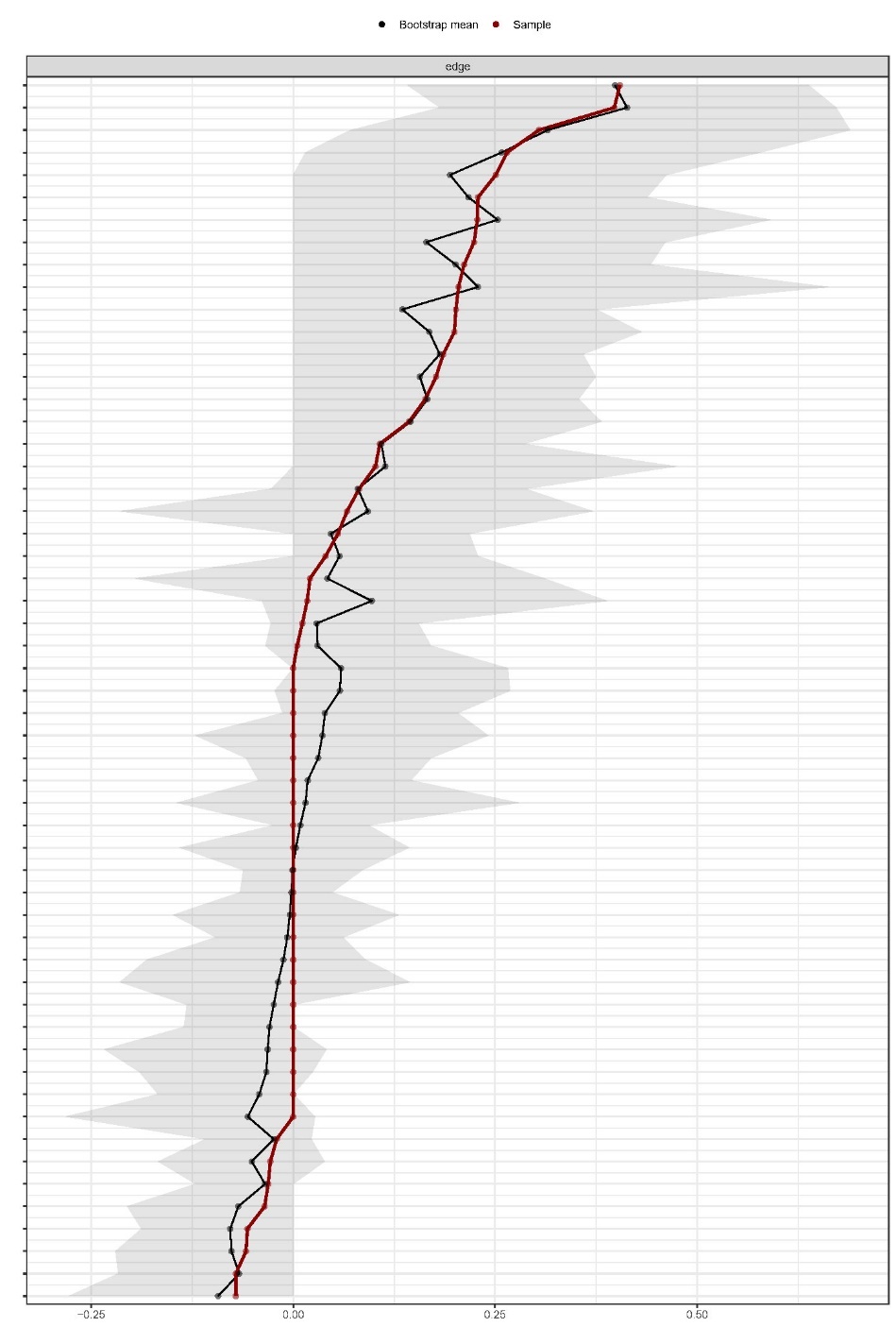


**Supplementary Figure S3** Bootstrapped confidence intervals of edge weights of the psychological resilience-social support-depressive symptom network

The black dots indicate the values of each edge weight, ordered from the highest to the lowest value. The gray area represents the 95% Confidence Intervals of edge weights, estimated with the non-parametric bootstrap procedure (by R *bootnet* package). Wide intervals suggest lower stability and narrow intervals mean higher stability.


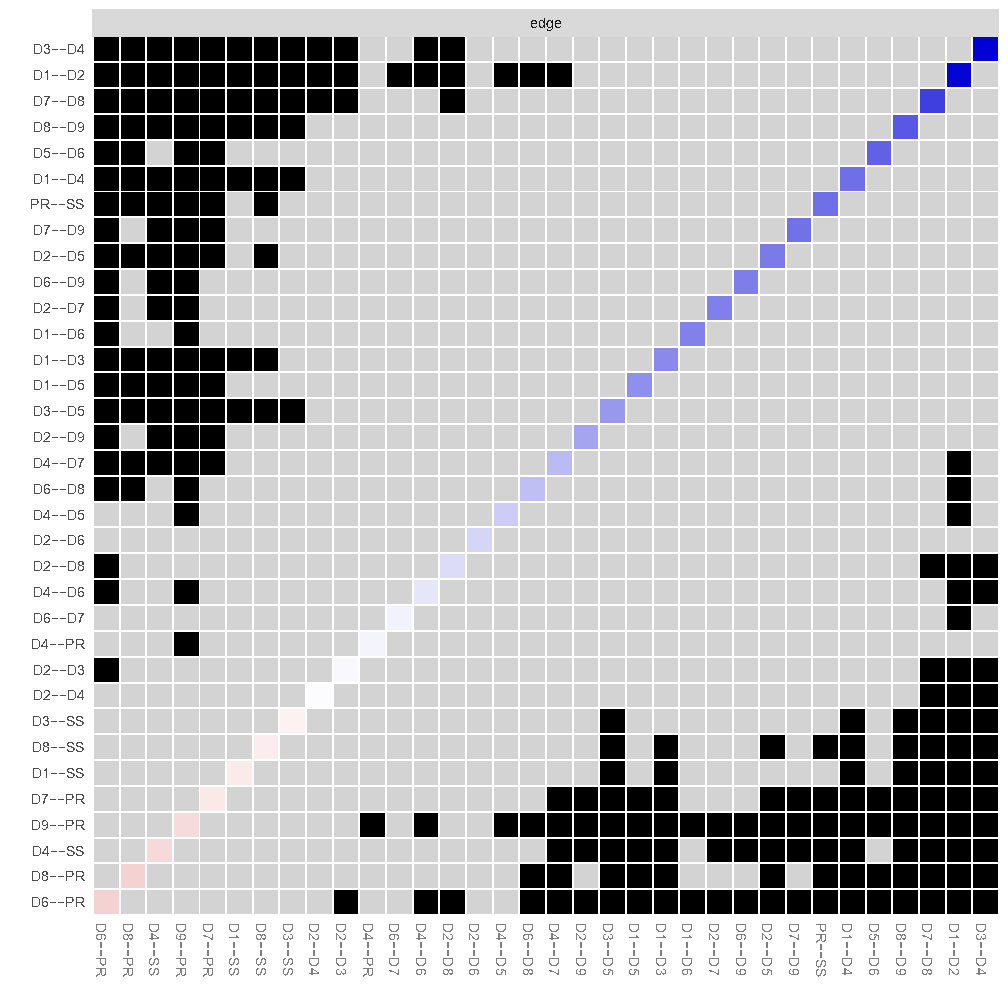


**Supplementary Figure S4** Estimation of edge weight difference by bootstrapped difference test of the psychological resilience-social support- depressive symptom network

Bootstrapped difference tests between edge weights in the network. Gray boxes suggest edges that do not differ significantly from one-another. Black boxes indicate edges with significant difference from one another (α = 0.05). Blue boxes in the edge-weight diagram indicate positive correlations.

**
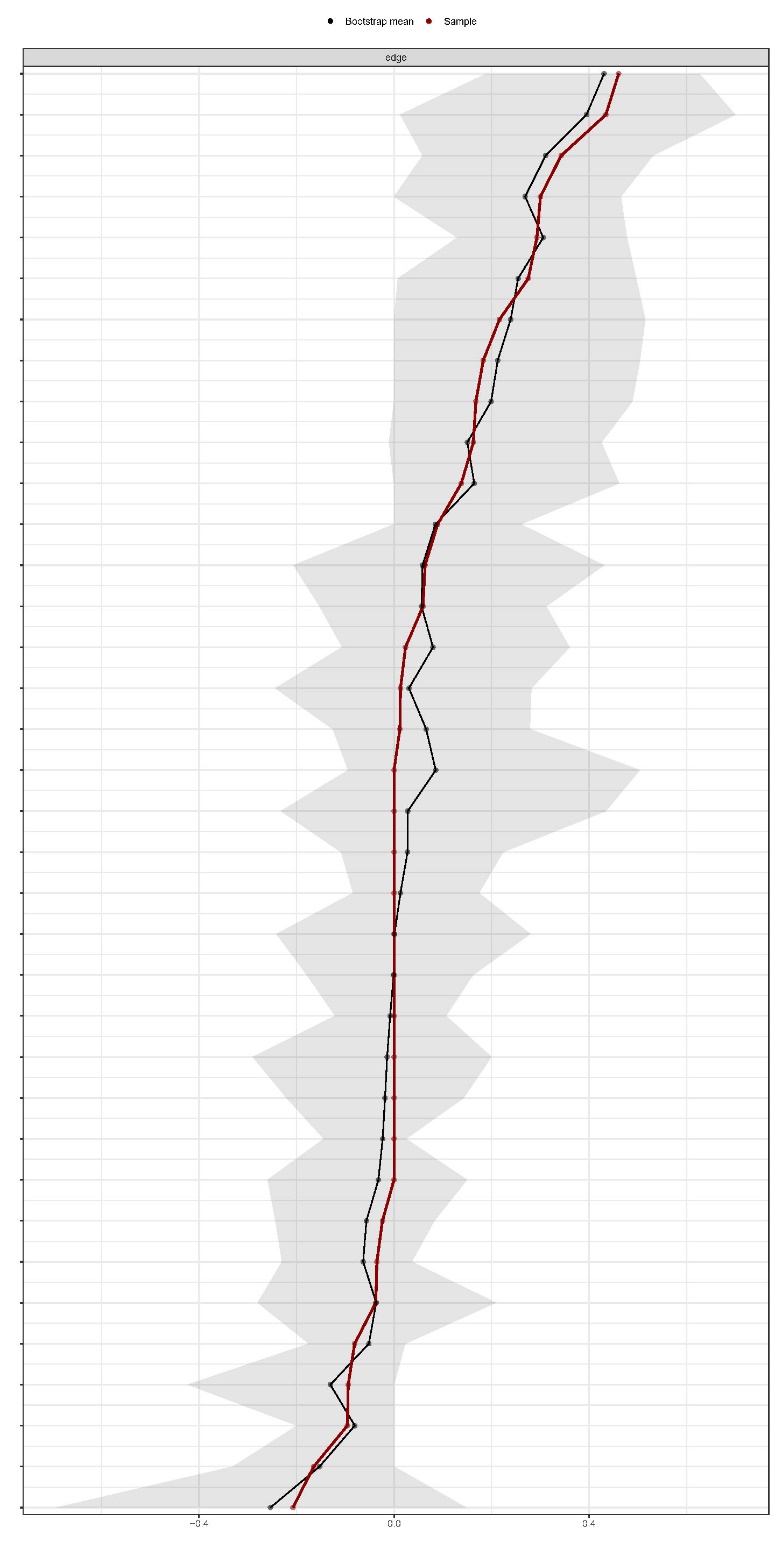
**

**Supplementary Figure S5** Bootstrapped confidence intervals of edge weights of the psychological resilience-social support-anxiety symptom network

The black dots indicate the values of each edge weight, ordered from the highest to the lowest value. The gray area represents the 95% Confidence Intervals of edge weights, estimated with the non-parametric bootstrap procedure (by R *bootnet* package). Wide intervals suggest lower stability and narrow intervals mean higher stability

**
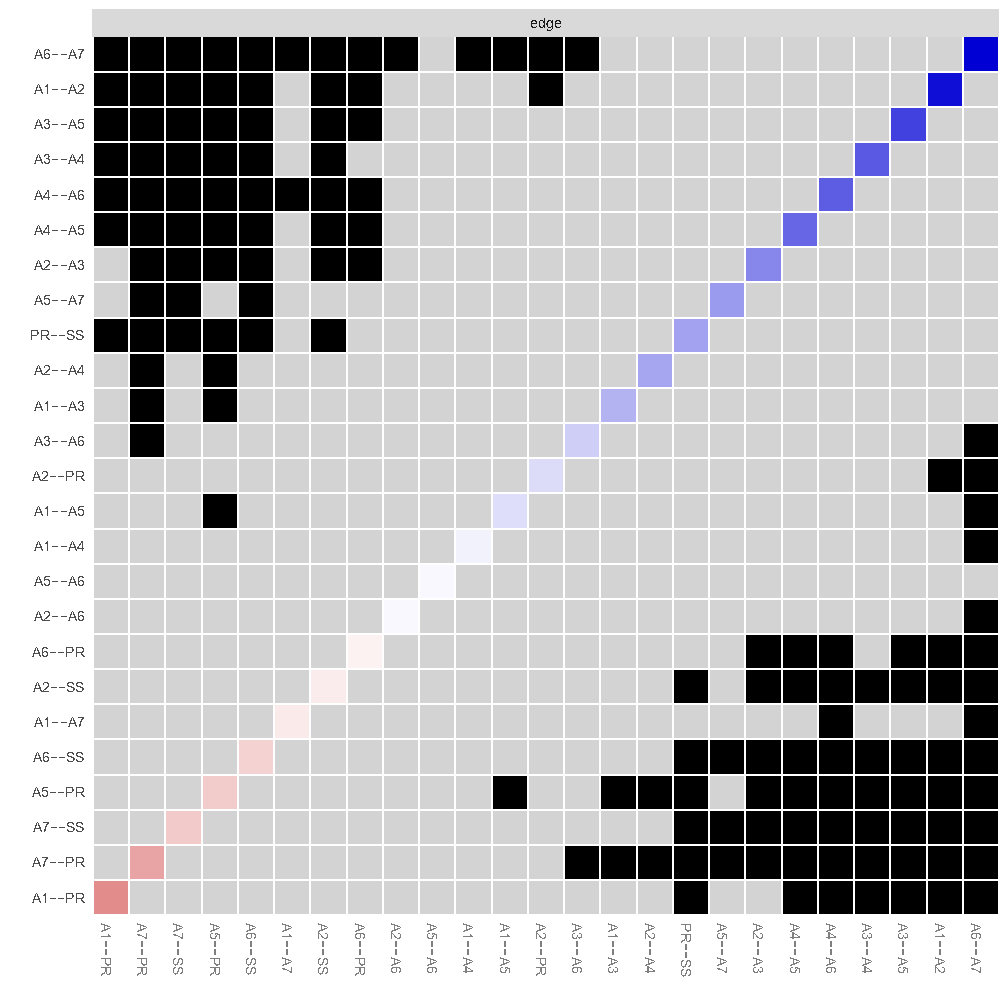
**

**Supplementary Figure S6** Estimation of edge weight difference by bootstrapped difference test of the psychological resilience-social support- anxiety symptom network

Bootstrapped difference tests between edge weights in the network. Gray boxes suggest edges that do not differ significantly from one-another. Black boxes indicate edges with significant difference from one another (α = 0.05). Blue boxes in the edge-weight diagram indicate positive correlations.
